# INO80 haploinsufficiency inhibits colon cancer tumorigenesis via replication stress-induced apoptosis

**DOI:** 10.18632/oncotarget.22984

**Published:** 2017-12-06

**Authors:** Shin-Ai Lee, Han-Sae Lee, Shin-Kyoung Hur, Sang Won Kang, Goo Taeg Oh, Daekee Lee, Jongbum Kwon

**Affiliations:** ^1^ Department of Life Science, Ewha Womans University, Seodaemun-gu, Seoul, 03760, Korea; ^2^ The Research Center for Cellular Homeostasis, Ewha Womans University, Seodaemun-gu, Seoul, 03760, Korea

**Keywords:** INO80 chromatin remodeling complex, colon cancer, APC Min mouse model, replication stress, apoptosis

## Abstract

The INO80 chromatin-remodeling complex performs functions in many chromosomal processes that are crucial for genome stability, such as DNA replication and stalled replication fork recovery. Although these functions suggest that INO80 acts as a tumor suppressor, its specific role in tumorigenesis has remained obscure. Here, we show that a haploinsufficient mutation of Ino80, the catalytic ATPase of the INO80 complex, decreased intestinal adenomatous polyps and increased survival in an Apc^min/+^ mouse model of colon cancer. Experiments using tumors obtained from Apc^min/+^ mice and cells from human colon cancers showed that this Ino80 defect induced stalled replication forks, the concomitant activation of ATR-Chk1 signaling and an increase in apoptosis, suggesting that Ino80 haploinsufficiency inhibited colon cancer tumorigenesis by activating replication stress-induced ATR-Chk1 signaling to increase apoptosis. Importantly, in human colon cancer, we observed that the INO80 subunits were frequently present in high copy numbers and exhibited a high rate of amplification and increased protein expression. These results show that in contrast to our original prediction that INO80 acts as a tumor suppressor, INO80 actually functions oncogenically to promote colon tumorigenesis. INO80 therefore represents a novel therapeutic target in colon cancer. The results of this study also reinforce the emerging notion that while genomic instability can promote tumorigenesis, in certain genetic contexts, it can also act as a tumor suppressor.

## INTRODUCTION

INO80 belongs to the INO80 subfamily of the ATP-dependent chromatin-remodeling complex and functions as a multi-protein complex that is comprised of at least 15 subunits, including the evolutionarily conserved Ino80 ATPase [1, 2]. INO80 modulates chromatin structure by mobilizing nucleosomes at the expense of the energy produced by ATP hydrolysis. Studies performed over the last decade indicate that INO80 plays important role in many chromosomal processes that are crucial for genome integrity. These processes include DNA damage repair, telomere regulation, and chromosome segregation, and it is particularly important in DNA replication under both non-stressed and stressed conditions [3–11]. Consistent with these functions, INO80 is also important to maintaining genome stability, and inactivating or depleting INO80 results in aneuploidy and chromosome structural abnormalities [12–15]. Because there is a causal association between genomic instability and tumorigenesis [16], these findings suggest that INO80 may function as a tumor suppressor.

The data presented in recent studies have provided evidence that INO80 is associated with tumorigenesis. BRCA1-associated protein 1 (BAP1), a nuclear deubiquitinase with tumor suppressor activity, stabilizes and recruits INO80 to replication forks for efficient DNA replication, thereby ensuring genome stability [6]. Importantly, Ino80 levels are often reduced in BAP1-null mesothelioma cells, which lack a BAP1-mediated Ino80 stabilization mechanism, and downregulated in BAP1-defective cancer cells in mesothelioma patients [6]. These results suggest that INO80 may function downstream of BAP1 to suppress tumorigenesis in mesothelioma. Additionally, the organismal functions of INO80 have been studied using a gene-targeting approach. For example, inducing a homozygous mutation in *Ino80* resulted in embryonic lethality, whereas *Ino80*-heterozygous mice exhibited no obvious phenotype [6, 17, 18], indicating that INO80 plays an essential role in mouse embryogenesis. While inducing the conditional deletion of *Ino80* in adult mice had no effect on spontaneous tumor incidence, inducing *Ino80* haploinsufficiency in a *p53*^−/−^ background impacted cancer development by shifting the tumor type from lymphoma to sarcoma, which was suggested to be due to increased genomic instability and consequent defects in DNA repair and gene transcription involved in tumorigenesis [17].

Colorectal cancer is thought to be causally associated with genomic instabilities because it exhibits aneuploidy, microsatellite instability and chromosome structural abnormalities during the very early stages of tumorigenesis [19]. Adenomatous polyposis coli (Apc) is one of the factors that plays a crucial role in tumorigenesis in colorectal cancer. Apc triggers the proteolytic degradation of β-catenin by inducing its phosphorylation and subsequent ubiquitination, and defects in the function of Apc result in the nuclear accumulation of β-catenin and the activation of target genes that stimulate the cell cycle [20, 21]. Genomic instability can generate mutations in key genes, such as *Apc* and *p53*, which are critical for regulating cell proliferation and cell cycle checkpoints, and this instability can eventually lead to the development of colorectal cancer [19]. The multiple intestinal neoplasia (min) mouse carries a truncating mutation in the *Apc* gene (*Apc*^min/+^) that causes adenomatous polyps to develop in the small intestine and colon. This line of mice serves as the most widely used model for intestinal neoplasia [22, 23].

In the present study, we investigated the impact of INO80 on tumorigenesis using colon cancer as a model case. Because the homozygous deletion of *Ino80* results in embryonic lethality, we introduced an *Ino80* haploinsufficiency mutation in an *Apc* min background by crossing *Apc*^min/+^ and *Ino80*+/− mice. Whereas we had originally predicted that INO80 would function as a tumor suppressor, we found that INO80 actually acted oncogenically in colon cancer tumorigenesis in that Ino80 haploinsufficiency inhibited intestinal tumors in the *Apc*^min/+^ mice. Our exploration of the mechanism underlying this effect suggested that Ino80 haploinsufficiency inhibited colon cancer tumorigenesis by activating replication stress-induced ATR-Chk1 signaling to increase apoptosis. Finally, we also found that INO80 is upregulated in human colon cancer, indicating that our findings have clinical significance.

## RESULTS

### Ino80 haploinsufficiency inhibits intestinal tumorigenesis and increases survival in Apc^min/+^ mice

**Figure 1:**
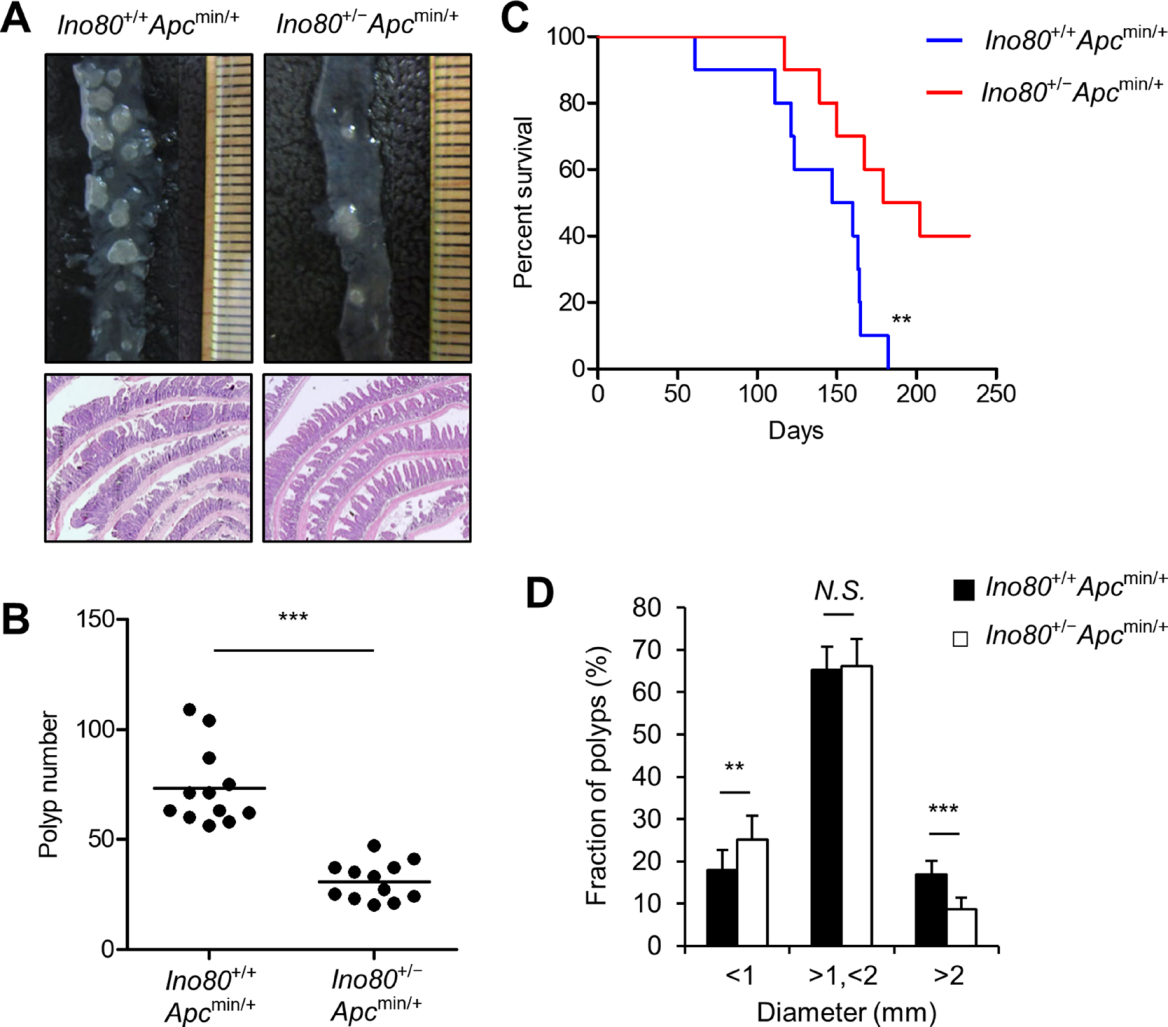
*Ino80* haploinsufficiency inhibits intestinal tumorigenesis in *Apc*^min/+^ mice (**A**) *Ino80*^+/+^*Apc*^min/+^ and *Ino80*^+/−^*Apc*^min/+^mice were killed when they were 100 days old. The intestines were isolated to count the number of polyps. Representative microscopic images (top) and hematoxylin and eosin-stained samples (bottom) of small intestinal polyps are shown. Magnification, x40. (**B**) The number of small intestinal polyps in the indicated mouse lines (*n* = 12 for each group) was plotted as a graph in which each dot represents the total polyp number in a single mouse. The horizontal bar indicates the mean value. The *p* values were determined using the Wilcoxon rank-sum test. (**C**) Kaplan–Meier survival curve comparing survival in the indicated mice (median survival: *Ino80*^+/+^*Apc*^min/+^=153.5 days *vs Ino80*^+/−^*Apc*^min/+^ = 190.5 days; *n* = 10 per group). The *p* value was determined using the log-rank test. (**D**) The size distribution of small intestinal polyps obtained from the indicated mice. The data were obtained from the results shown in (A). ^**^p < 0.01; ^***^p < 0.001. *N.S.*, not significant. Error bars, mean ± SD.

To determine whether INO80 is involved in colon cancer tumorigenesis, we crossbred *Ino80*^+/−^ mice with *Apc*^min/+^ mice and analyzed the intestinal polyps of the resulting offspring at the age of 100 days. The genotypes of the mice were determined using PCR as previously described [6]. *Ino80*^+/+^*Apc*^+/+^ and *Ino80*^+/−^*Apc*^+/+^ mice had virtually no detectable polyps in the intestines (data not shown), indicating that the reduction in Ino80 did not affect intestinal tumorigenesis. *Ino80*^+/+^*Apc*^min/+^ and *Ino80*^+/−^*Apc*^min/+^ mice were born in the same ratios, and there was no apparent difference in gross phenotype or growth between these lines up until approximately 100 days after birth, when the mice were sacrificed for polyp analysis (data not shown). We next compared *Ino80*^+/+^*Apc*^min/+^ and *Ino80*^+/−^*Apc*^min/+^ mice with regard to the development of adenomatous polyps in the intestines. The *Ino80*^+/+^*Apc*^min/+^ mice developed an average of 74 adenomatous polyps in the small intestine and a few in the colon, and these results were consistent with those previously reported for *Apc*^min/+^ mice [23]. However, we unexpectedly found that the average number of adenomatous polyps in the small intestines of the *Ino80*^+/−^*Apc*^min/+^ mice was approximately 50% lower than the number in the *Ino80*^+/+^*Apc*^min/+^ mice (Figure 1A and 1B), indicating that haploinsufficiency in *Ino80* inhibited the development of small intestinal tumors. A similar magnitude of tumor inhibition was observed in the colons in the *Ino80*^+/−^*Apc*^min/+^ mice (Supplementary Figure 1A and 1B). Consistent with these results, the survival rate was significantly higher in the *Ino80*^+/−^*Apc*^min/+^ mice than in the *Ino80*^+/+^*Apc*^min/+^ mice (Figure 1C).

An analysis of polyp size showed that a large portion of the polyps in the small intestine was 1–2 mm in diameter and their frequency was not significantly different between the *Ino80*^+/+^*Apc*^min/+^ and *Ino80*^+/−^*Apc*^min/+^ mice (Figure 1D). The size distribution of the remaining polyps was such that the percentage of the polyps smaller than 1 mm was higher whereas the percentage of the polyps larger than 2 mm was lower in the *Ino80*^+/−^*Apc*^min/+^ mice than the *Ino80*^+/+^*Apc*^min/+^ mice (Figure 1D). Thus, haploinsufficiency in *Ino80* has an impact on both tumor number and tumor size.

### Ino80 haploinsufficiency increases apoptosis in intestinal tumor cells in Apc^min/+^ mice

**Figure 2:**
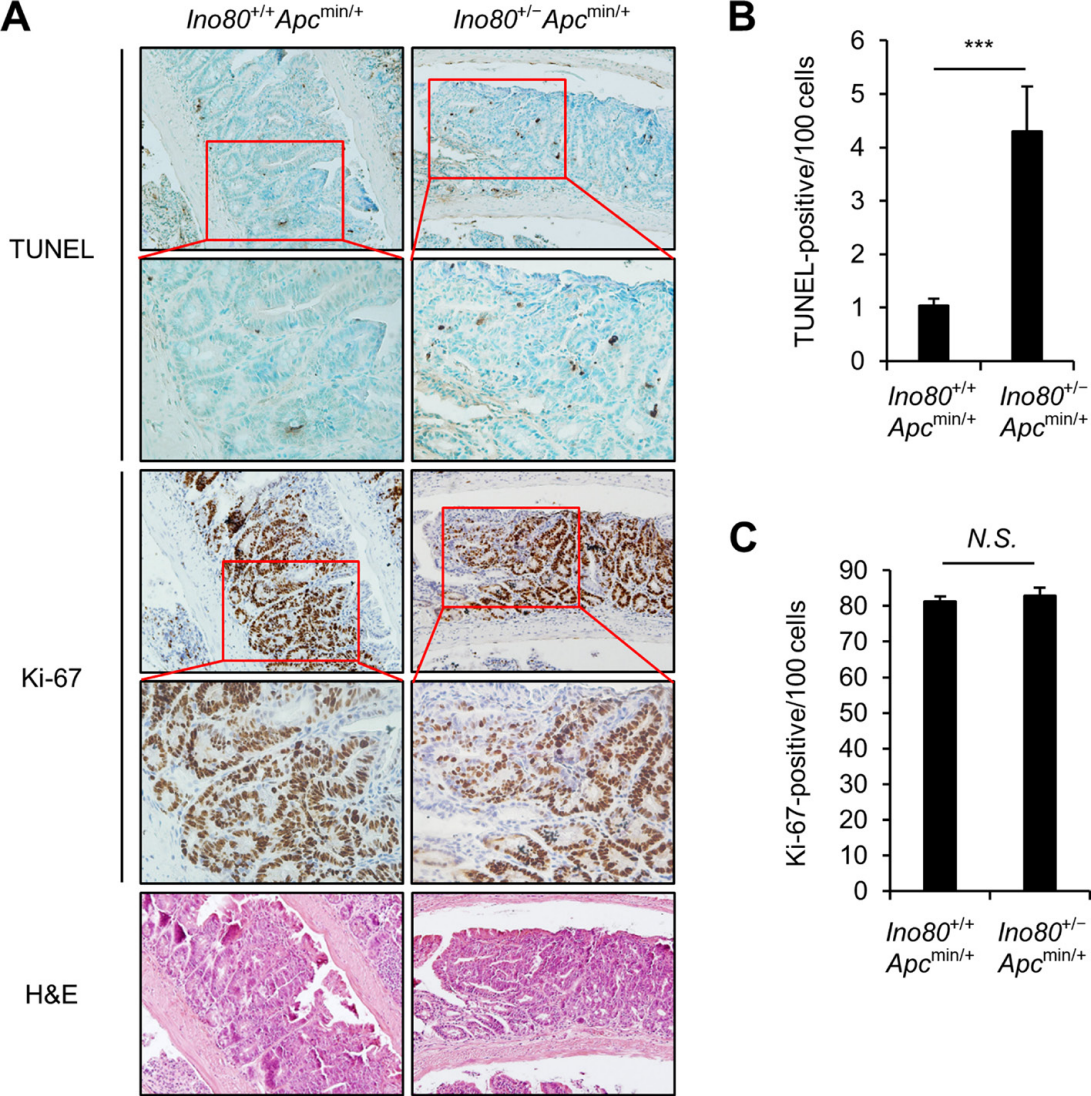
*Ino80* haploinsufficiency increases apoptosis in intestinal tumor cells in *Apc*^min/+^ mice (**A**) Representative results of TUNEL, Ki-67, and hematoxylin and eosin staining in size-matched small intestinal polyps obtained from the indicated mouse lines when the mice were 100 days old. Five tumoral regions per mouse were randomly selected as regions of interest (ROI) to calculate the average number of TUNEL- and Ki-67-positive cells. The results of our analysis of one representative tumor out of those obtained from four mice that exhibited similar results are shown. Original magnification, **×** 200; enlargement, x400. (**B**) The apoptotic index for small intestinal tumors in the indicated mice is represented as the percentage of TUNEL-positive cancer cells. Error bars, mean ± SD (*n* = 4). (**C**) The proliferation index in small intestinal tumors obtained from the indicated mice is represented as the percentage of Ki-67-positive cancer cells. Error bars, mean ± SD (*n* = 4).

Next, we investigated the mechanisms by which *Ino80* haploinsufficiency inhibits tumorigenesis in *Apc*^min/+^ mice. For these experiments, we focused on small intestinal tumors. We rationalized that tumor can be inhibited by either increasing apoptosis or impeding proliferation in tumor cells or both. First, we performed TUNEL assays using paraffin-embedded sections of tumors obtained from *Ino80*^+/+^*Apc*^min/+^ and *Ino80*^+/−^*Apc*^min/+^ mice. The average number of TUNEL-positive cancer cells in the small intestinal tumors of *Ino80*^+/−^*Apc*^min/+^ mice was approximately four times higher than the number in tumors obtained from *Ino80*^+/+^*Apc*^min/+^ mice (Figure 2A and 2B), indicating that there was a higher rate of apoptosis in the tumors in the *Ino80*^+/−^*Apc*^min/+^ mice. We next stained the paraffin-embedded sections of tumors obtained from the *Ino80*^+/+^*Apc*^min/+^ and *Ino80*^+/−^*Apc*^min/+^ mice with an antibody against Ki-67, which is a marker of proliferation. There was no difference between the percentages of Ki-67-positive cancer cells in the small intestinal tumors obtained from the two lines of mice (Figure 2A and 2C). These results suggest that the decreased intestinal tumorigenesis in the *Apc*^min/+^ mice with Ino80 reduction is possibly due to an increase in apoptosis rather than a decrease in the proliferation of tumor cells.

### Ino80 knockdown increases apoptosis in human colon cancer cells

**Figure 3:**
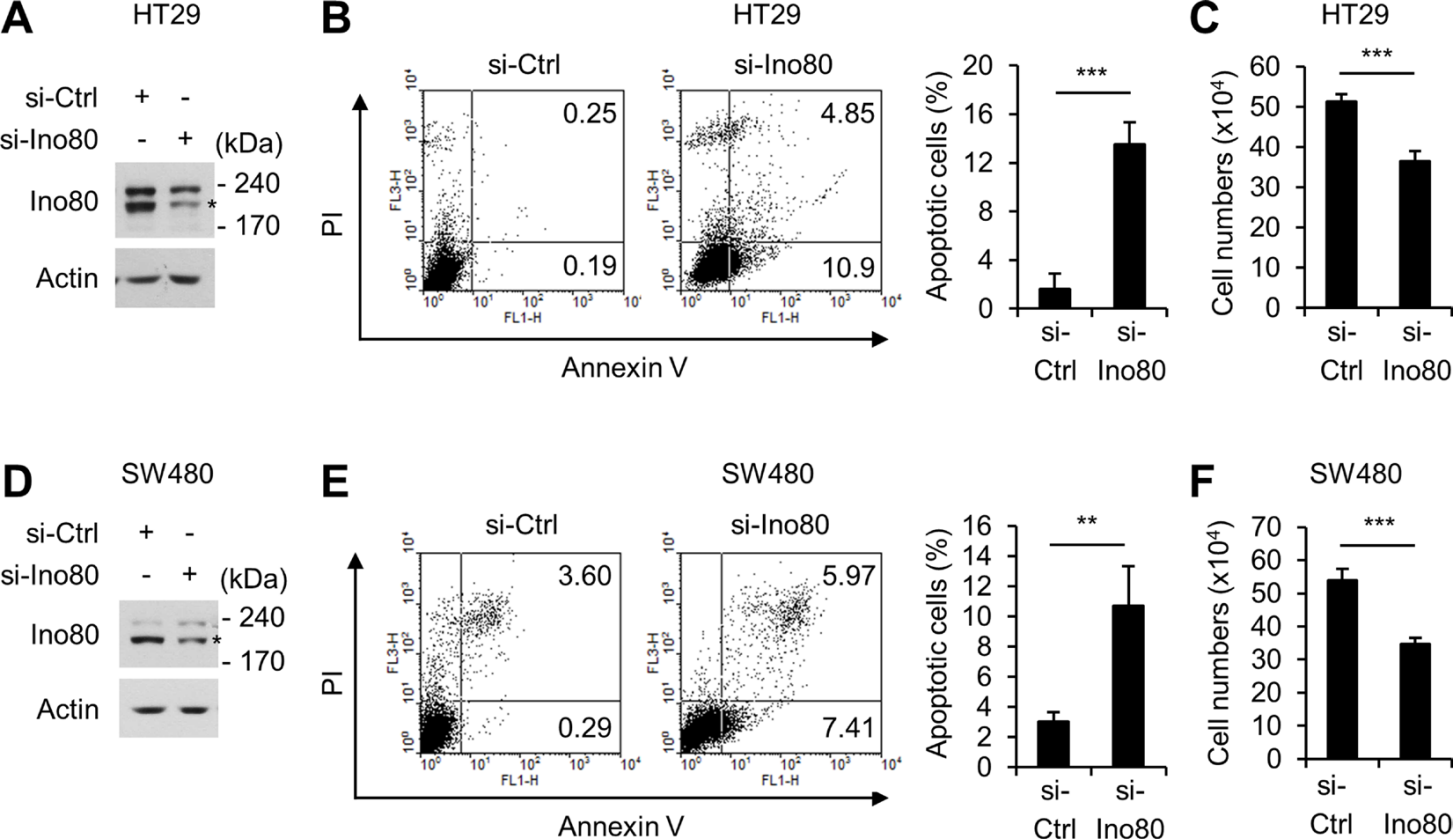
Ino80 knockdown increases apoptosis in human colon cancer cells (**A**) Immunoblot analysis of Ino80 expression in HT29 cells following a 72-h transfection with control or Ino80-specific siRNAs. (**B**) An increase in apoptosis was observed in HT29 cells following Ino80 knockdown. (left) After the cells were transfected as in (A), they were subjected to Annexin V and PI staining followed by FACS analysis. A representative result is shown as the percentage of Annexin V-positive apoptotic cells. (right) The results of an apoptosis assay are shown as a graph. Error bars, mean ± SD of three independent experiments. (**C**) After the cells were transfected as in (A), the number of viable cells was determined using a trypan blue exclusion assay in triplicate. Error bars, mean ± SD of three independent experiments. (**D**–**F**) The results of experiments performed using SW480 under similar conditions to those performed in (A-C) are shown. Ino80 band was indicated by star mark in (A) and (D).

We next sought to determine the mechanisms by which the reduction in Ino80 increased apoptosis in the intestinal tumors of *Apc*^min/+^ mice. In these experiments, we used two human colon cells, HT29 and SW480, which mimic the tumor cells in *Apc*^min/+^ mice in that they harbor inactivating mutations in *Apc* [24]. In HT29 cells, knocking down Ino80 using a specific small interfering RNA resulted in a large increase in Annexin-V-positive apoptotic cells (Figure 3A and 3B). Consistent with these results, trypan blue exclusion assays showed that Ino80 knockdown resulted in a decrease in cell viability (Figure 3C). We obtained virtually the same results when we performed a similar experiment using SW480 cells (Figure 3D–3F). Therefore, we concluded that knocking down Ino80 increases apoptosis in human colon cancer cells.

### Ino80 knockdown increases stalled replication forks in human colon cancer cells

**Figure 4:**
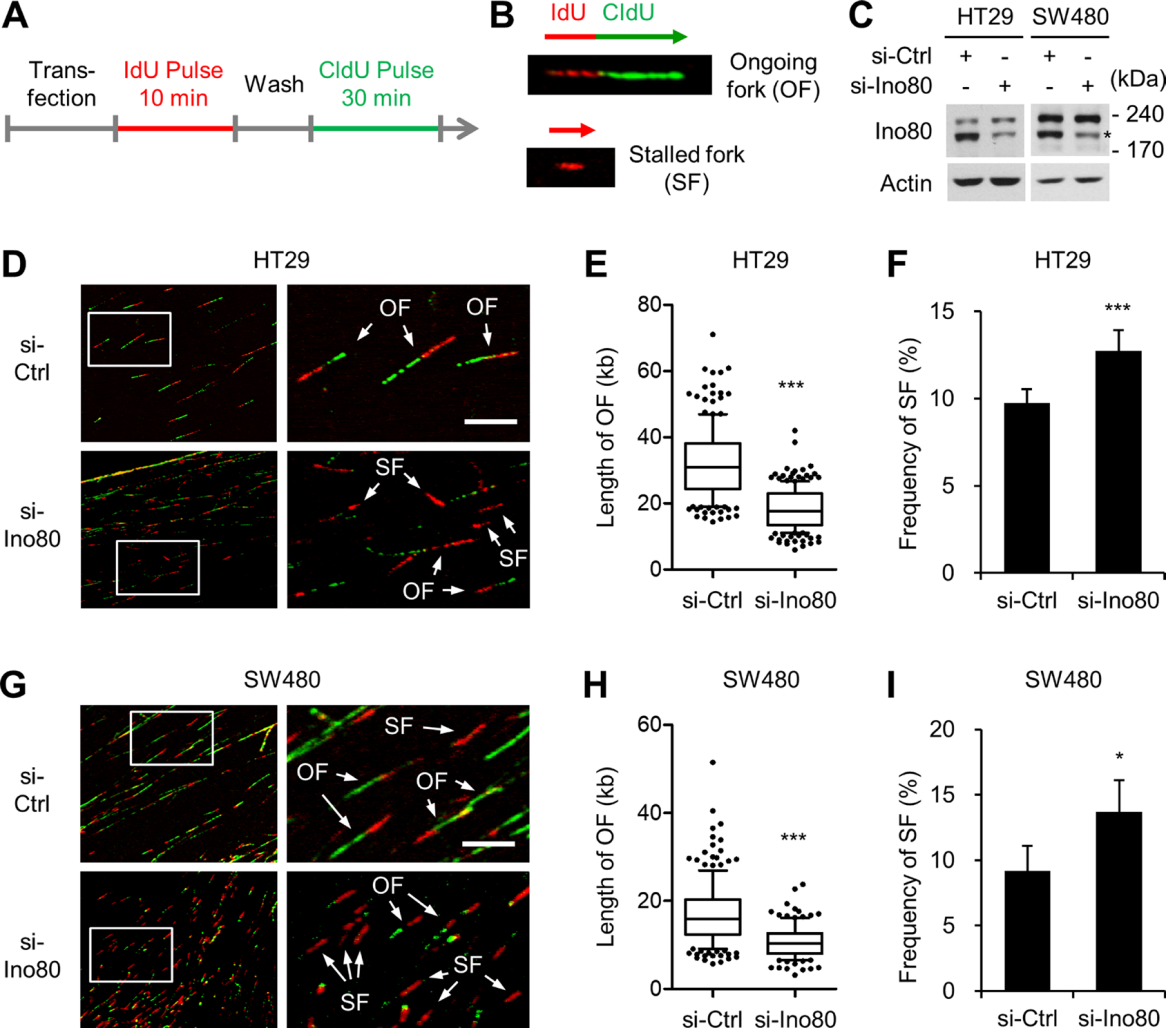
Ino80 knockdown decreases replication rates and increases stalled replication forks in human colon cancer cells (**A**) The experimental strategy used for the DNA fiber assay. (**B**) Examples of DNA fiber with IdU/CldU incorporation showing a representative ongoing fork (OF) and DNA fiber with CldU incorporation only showing a representative stalled fork (SF). (**C**) siRNA knockdown of Ino80 in HT29 and SW480 cells. Ino80 band was indicated by star mark. (**D**) HT29 cells that were transfected with siRNA were subjected to DNA fiber assays. Representative fiber images are shown. The selected areas are shown magnified for visual enhancement of OF and SF. (**E**) The tract lengths of OF were determined from the results shown in (D) and are illustrated as a box and whisker plot. The data were derived from one of three independent experiments with similar results in which at least 150 fibers were analyzed per experiment. Whiskers indicate the 10^th^ and 90^th^ percentiles. Scale bar, 10 mm. ^***^p < 0.001, Mann-Whitney *U* test. (**F**) The frequency of SF was determined from the results shown in (D) and is illustrated as a graph. ^***^p < 0.001; Error bars, mean ± SD of three independent experiments. (**G**–**I**) Experiments similar to those shown in (D-F) were performed using SW480 cells. ^***^p < 0.001, Mann-Whitney *U* test. ^*^p < 0.05; Error bars, mean ± SD of three independent experiments.

Studies using HeLa human cervical cancer cells and PC3 human prostate cancer cells have shown that INO80 performs a role in replication fork progression during normal DNA synthesis and in the process of recovering from replication stress induced by treatment with hydroxyurea [6, 12, 25]. We therefore used DNA fiber-labeling assays to determine whether INO80 performs these functions in human colon cancer cells. The DNA in actively growing HT29 and SW480 cells was labelled consecutively with iodo-deoxyuridine (IdU) and chloro-deoxyuridine (CldU). The DNA was extended and then visualized using immunofluorescence microscopy to detect *de novo* DNA synthesis at the level of individual DNA molecules (Figure 4A). This strategy allowed us to distinguish between different replication intermediates, such as ongoing forks (IdU/CldU incorporation) and stalled forks (IdU incorporation only) (Figure 4B). Knocking down Ino80 using siRNA (Figure 4C) decreased the track length of ongoing forks in both HT29 (Figure 4D and 4E) and SW480 (Figure 4G and 4H) cells, indicating that as in HeLa and PC cells, INO80 plays a role in normal replication fork progression in colon cancer cells. Notably, in both HT29 (Figure 4D and 4F) and SW480 (Figure 4G and 4I) cells, there was a small fraction (approximately 10%) of spontaneous stalled forks (indicated by red only). This percentage was significantly increased by Ino80 knockdown. These results show that INO80 is required to suppress stalled replication fork generation in normal cultures of human colon cancer cells.

### Ino80 knockdown activates ATR-Chk1 signaling in human colon cancer cells

**Figure 5:**
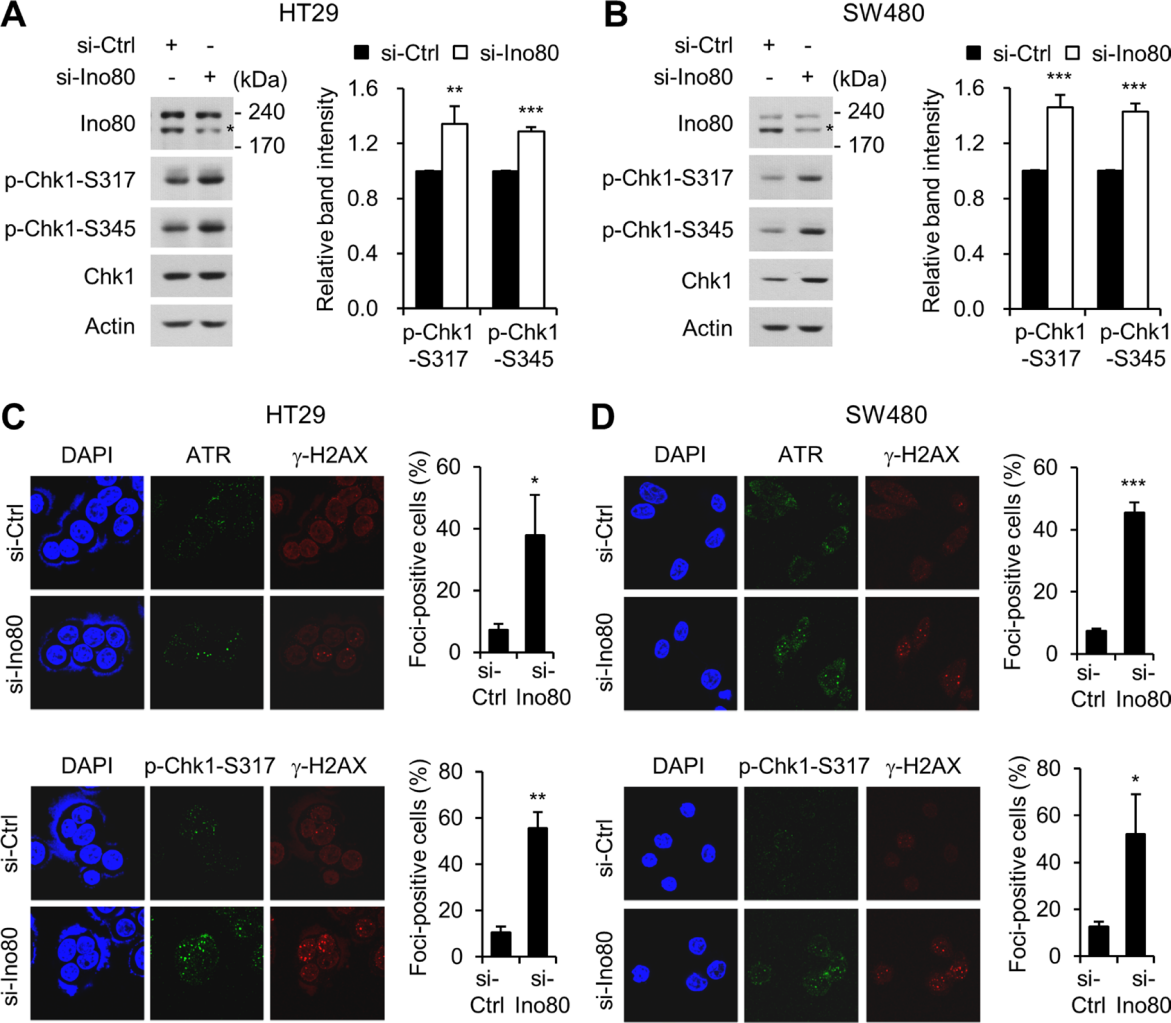
*Ino80* knockdown activates ATR-Chk1 signaling in human colon cancer cells (**A**) HT29 cells were transfected with non-specific or Ino80-specific siRNAs, and whole-cell lysates were analyzed using immunoblotting with the indicated antibodies. Band intensities were quantified using Image Gauge software (left). The normalized ratios (phosphorylated/total) of CHK1 activity are shown as a graph (right). A representative result from three independent experiments is shown. (**B**) SW480 cells were subjected to the same experiments as in (A). Ino80 band was indicated by star mark on the gels in (A) and (B). (**C**) The effect of Ino80 knockdown on the replication stress-induced ATR-Chk1 signaling in HT29 cells. After transfection was performed as in (A), HT29 cells were double-stained with either anti-ATR (top) or anti-p-CHK1 (bottom) in addition to anti-γ-H2AX antibodies. Confocal images were captured, and more than 50 cells were scored per condition. The results are presented as the percent of foci-positive cells and illustrated as a graph to the right of the representative images. (**D**) SW480 cells were subjected to the same experiments as in (C). ^*^p < 0.05; ^**^p < 0.01; ^***^p < 0.001; Error bars, mean ± SD of three independent experiments.

Replication stress generates abnormal replication fork structures, resulting in single-stranded DNA. The generation of these structures activates ATR (ataxia-telangiectasia and Rad3 related), which in turn activate the downstream effector Chk1 (checkpoint kinase 1) by phosphorylating it at Ser-317 and Ser-345. ATM-Chk1 signaling leads to the activation of the cell cycle checkpoint, the stabilization and restarting of stalled replication forks, and cell death via apoptosis under conditions involving too much replication stress [26, 27]. Because we found that Ino80 depletion induced stalled replication forks, we next asked whether Ino80 activates ATR-Chk1 signaling in human colon cancer cells. We first addressed this question using immunoblot analysis. We found that depleting Ino80 in HT29 and SW480 cells resulted in the activation of ATR-Chk1 signaling, as indicated by an increase in the phosphorylation of Chk1 at Ser-317 and Ser-345 (Figure 5A and 5B). We confirmed these results by analyzing ATR and phospho-Chk1 foci formation using immunofluorescence microscopy. Because DNA replication is often accompanied by γ-H2AX foci formation at stalled replication forks [28], we also detected γ-H2AX foci as a marker of stalled forks. We found that Ino80 depletion in HT29 and SW480 cells substantially increased both ATR and phospho-Chk1 foci formation (Figure 5C and 5D). These results show that a defect in Ino80 resulted in the activation of ATR-Chk1 signaling in human colon cancer cells.

### Ino80 haploinsufficiency enhances Chk1 activation in intestinal tumors in Apcmin/+ mice

**Figure 6:**
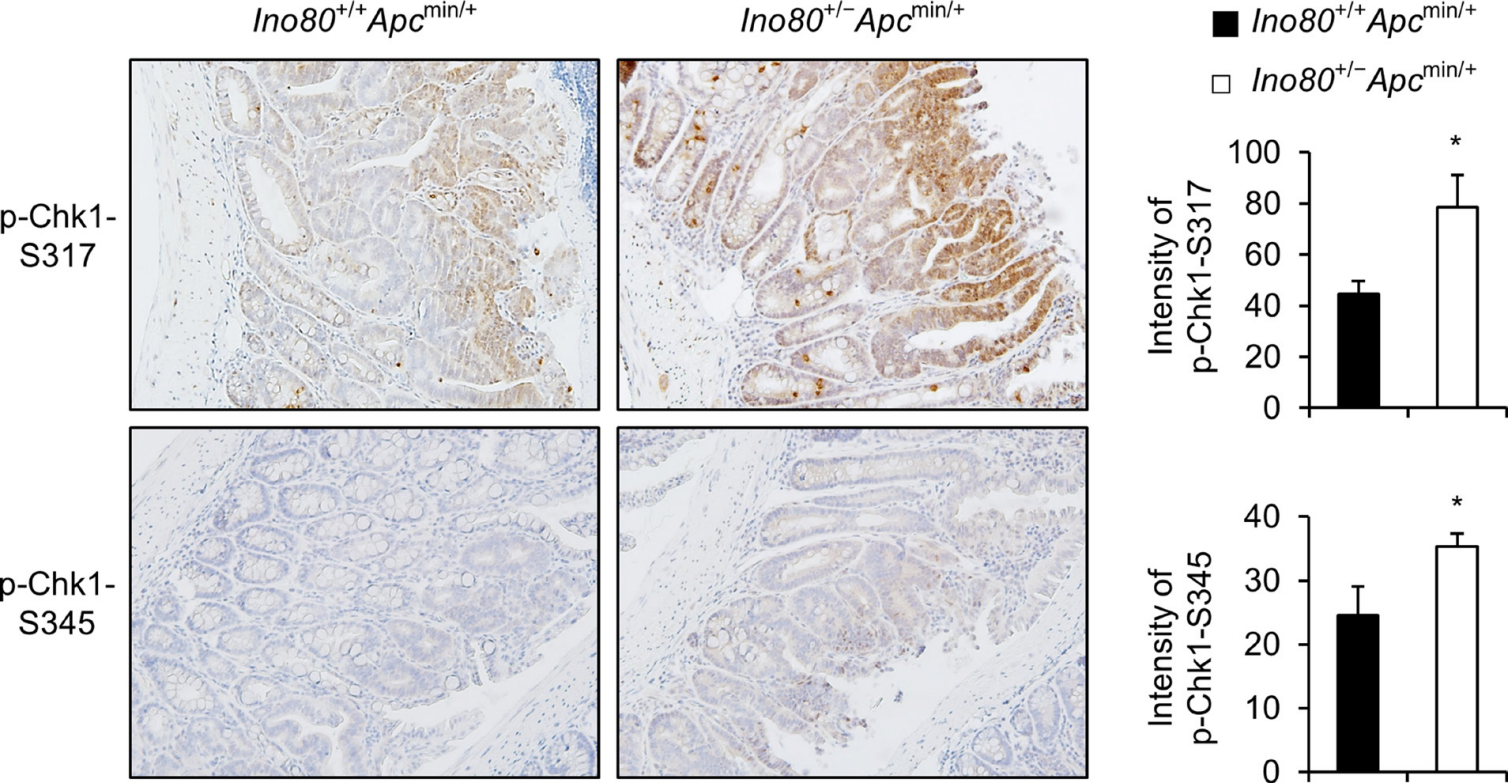
*Ino80* haploinsufficiency activates Chk1 in intestinal tumors in *Apc*^min/+^ mice Representative results are shown for p-Chk1-S317 and p-Chk1-S345 immunostaining in size-matched small intestinal tumors obtained from the indicated mice when they were 100 days old. Original magnification, × 200. Five tumoral regions per mouse were designated regions of interest (ROI), and their mean immunostaining intensities were quantified using TissueFAXS. The quantitation data are illustrated as a graph and shown next to the corresponding immunostaining images. Error bars, mean ± SD of results from three different mice.

Next, we sought to determine whether Chk1 is activated at a higher rate when Ino80 is reduced in intestinal tumors in *Apc*^min/+^ mice. We stained intestinal tumors obtained from *Ino80*^+/+^*Apc*^min/+^ and *Ino80*^+/−^*Apc*^min/+^ mice using antibodies against phopsho-Chk1-Ser-317 or phospho-Chk1-Ser-345. We then compared the phosphorylation levels using a TissueFAXS analysis. The level of Chk1 phosphorylation at both sites was significantly higher in the tumors obtained from *Ino80*^+/−^*Apc*^min/+^ mice than in those obtained from *Ino80*^+/+^*Apc*^min/+^ mice (Figure 6). These results indicate that *Ino80* haploinsufficiency accelerated Chk1 activation in the intestinal tumors of the *Apc*^min/+^ mice, which is consistent with the results of the Ino80 knockdown experiments performed using human colon cancer cells.

### INO80 is upregulated in human colon cancer

**Figure 7:**
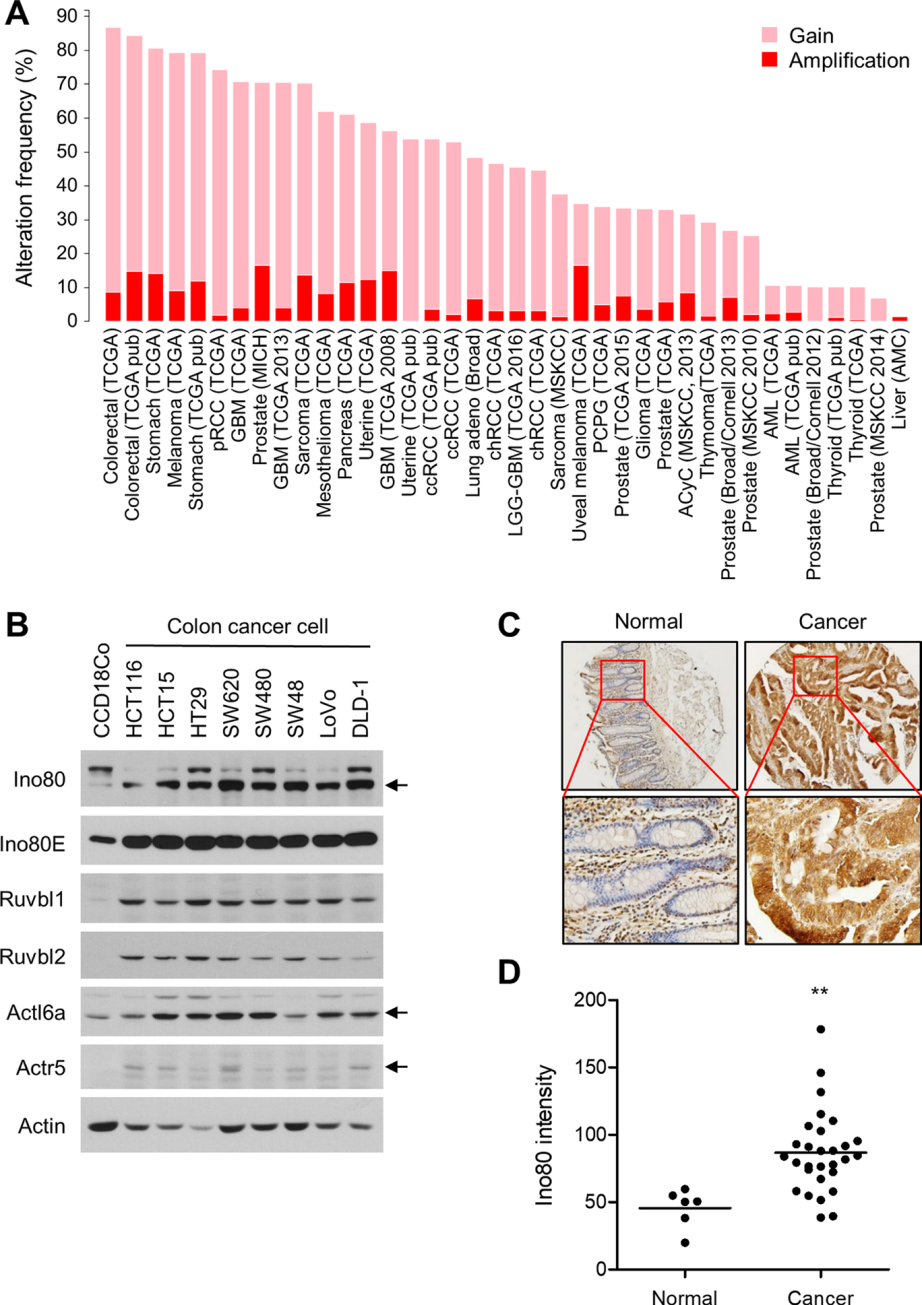
INO80 is upregulated in human colon cancer (**A**) The alteration frequency of INO80 subunits in various cancer types. The data shown were obtained from The Cancer Genome Atlas portal (http://www.cbioportal.org). Gain, one extra copy; Amplification, two or more extra copies (GISTIC 2.0, http://www.arrayserver.com/wiki/index.php?title=Gistic2_Call). (**B**) The protein levels of several INO80 subunits are shown for normal colon fibroblast cells (CCD18Co) and various colon cancer cells. Actin was used as the loading control. (**C**) Immunohistochemical staining for Ino80 in normal colon tissues (*n* = 6) and colon cancer tissues (*n* = 29) was performed using a tissue microarray (OriGene, CT565864). The mean immunostaining intensity of each tissue was quantified using TissueFAXS analysis. (**D**) The data from the experiments shown in (C) are illustrated as a dot plot. The horizontal lines indicate the average staining intensity in each group. ^**^p < 0.01, Mann-Whitney *U* test.

The results thus far suggest that INO80 acts oncogenically in intestinal tumorigenesis in *Apc*^min/+^ mice. Therefore, we sought to determine whether INO80 is upregulated in human colon cancer. First, we analyzed the frequency of alterations in INO80 subunit genes in a variety of cancers using The Cancer Genome Atlas (TCGA) database (http://cancergenome.nih.gov/). We found that INO80 subunits have a high frequency of gaining copy numbers and a high rate of amplification in many cancer types. Of these, colon cancer had the highest frequency (87%). However, different subunits were differentially affected (Figure 7A and Supplementary Figure 2). We next determined the expression level of Ino80 and several associated subunits in normal colon cells and a variety of colon cancer cells. Strikingly, we found that the levels of all of the INO80 subunits that were analyzed were higher in the colon cancer cells than in normal colon cells (Figure 7B). Furthermore, an analysis performed using a tissue microarray showed that the expression level of Ino80 was significantly higher in colon cancers than in normal colon tissues (Figure 7C and 7D, and Supplementary Figure 3). The Ino80 antibody was confirmed to be specific in the immunostaining of HT29 cells (Supplementary Figure 4). These data collectively demonstrate that INO80 is upregulated in colon cancer, which raises the possibility that INO80 plays an oncogenic role in colon cancer tumorigenesis.

## DISCUSSION

Here, we investigated whether INO80 is directly involved in tumorigenesis in colon cancer. We found that *Ino80* haploinsufficiency inhibited intestinal tumorigenesis in *Apc*^min/+^ mice. To study the mechanism involved in this process, we used tumors obtained from *Apc*^min/+^ mice and human colon cancer cells and showed that a deficit in Ino80 induced replication stress, leading to the ATR-mediated activation of Chk1 and increased apoptosis. These results suggest that *Ino80* haploinsufficiency inhibited colon cancer tumorigenesis by increasing replication stress-induced apoptosis. Importantly, in human colon cancers, INO80 subunits had a high frequency of gaining copy numbers and a high rate of amplification and there was an increase in the protein expression levels of these markers. These results collectively suggest that INO80 acts oncogenically to promote colon tumorigenesis. This remodeler is therefore a novel target for colon cancer therapy.

The finding that INO80 suppresses genomic instability led us to predict that INO80 would function as a tumor suppressor. However, our results showed that INO80 acts oncogenically in intestinal tumorigenesis in *Apc*^min/+^ mice. Genomic instability generally promotes tumorigenesis by causing mutations to accumulate in the genes responsible for tumor suppression [16]. A large number of studies in mouse models have targeted specific genes known to be involved in the cellular processes that are critical for genome stability, such as the DNA damage response and mitotic checkpoints, and their findings have supported this notion [29, 30]. For example, ablating the *ATM* DNA damage response gene was associated with a high incidence of lymphoid tumors in mice [31–33], and mice with a haploinsufficiency mutation in the *BubR1* mitotic checkpoint gene were prone to carcinogen-induced tumors [25, 34]. However, it has also been shown that genomic instability can inhibit tumorigenesis [35]. While mice with a haploinsufficiency mutation in the gene encoding CENP-E, a centromere-linked mitotic checkpoint protein that when defective produces aneuploidy, exhibited higher levels of spontaneous lymphomas and lung tumors, these mice also had lower rates of chemically or genetically induced tumor formation [36], indicating that genomic instability acted both oncogenically and as a tumor suppressor. The results presented in this study expand the oncogenic role of genomic instability and reinforce the developing concept that genomic instability can perform two competing functions in tumorigenesis.

Our findings are in line with those presented in other studies showing that genome stability proteins play oncogenic roles in *Apc*^min/+^ mice. Rao and colleagues explored whether aneuploidy induced by *BubR1* haploinsufficiency promoted intestinal tumorigenesis in *Apc*^min/+^ mice. They observed that *BubR1*^+/−^*Apc*^min/+^ mice developed 10 times more colon tumors than were observed in *Apc*^min/+^ mice, suggesting that BubR1 reduction-induced aneuploidy promoted colon tumorigenesis [37]. Interestingly, there were significantly fewer tumors in the small intestines of *BubR1*^+/−^*Apc*^min/+^ than were observed in *Apc*^min/+^ mice, and there was a higher rate of apoptosis in the intestinal tumors of *BubR1*^+/−^*Apc*^min/+^ mice, indicating that excessive aneuploidy promoted cell death and inhibited tumor growth [37]. Aneuploidy-mediated tumor suppression was also observed in a study performed using a mouse model of Down’s syndrome in which *Apc*^min/+^ mice harboring trisomy exhibited a significantly lower number of intestinal tumors than were observe in *Apc*^min/+^ mice [38].

Our data suggest that Ino80 insufficiency inhibited intestinal tumors in *Apc*^min/+^ mice by increasing the replication stress-induced activation of ATR-Chk1 signaling and thereby increasing apoptosis. The increased rate of apoptosis observed in the tumors of the *Ino80*^+/−^*Apc*^min/+^ mice could also have resulted from Ino80 deficiency-induced genomic instability itself, which can lead to the activation of apoptotic pathways other than those initiated by ATR-Chk1 signaling [39]. However, Ino80 deficiency may inhibit the development of tumors in *Apc*^min/+^ mice by impeding cell proliferation because depleting Ino80 has been shown to cause defective cell-cycle progression and slow growth in a variety of cell types [12]. However, this is unlikely to be the case in intestinal tumors in *Apc*^min/+^ mice because our data show that tumor cell proliferation was not affected by Ino80 deficiency. It should be noted that because INO80 is known to play a role in transcription regulation, it remains possible that Ino80 deficiency also inhibits intestinal tumors in *Apc*^min/+^ mice in part by inducing the dysregulation of gene expression. In support of this possibility, a recent study showed that INO80 promotes tumor growth in melanoma, non-small cell lung cancer and cervical cancer by activating the transcription of cancer-associated oncogenes [40–42].

The data presented in this and other studies have revealed that INO80 deficiency results in a diversity of cancer phenotypes. For example, it inhibited colon tumorigenesis by increasing apoptosis in the *Apc* min background, suppressed tumor growth in melanoma by affecting transcriptional regulation, and altered tumor types in the *p53* ^−/−^ background. It therefore appears that INO80 influences tumorigenesis via different mechanisms depending on the tumor type and genetic context. This might reflect the fact that it plays a diversity of functions in a wide variety of cellular processes. Further studies that explore additional cancer phenotypes in *Ino80*-ablated mice with different genetic backgrounds will increase our understanding of how INO80 impacts tumorigenesis.

## MATERIALS AND METHODS

### Mice and crosses

*Apc*^min/+^ mice with a C57BL/6 background were purchased from the Jackson Laboratory (Bar Harbor, ME). *Ino80* knockout mice were generated as previously described [6]. Male *Apc*^min/+^ mice were crossed with female *Ino80*^+/−^ mice to generate *Ino80*^+/−^*Apc*^min/+^ mice. All mating was performed in a clean, vented-rack room at the Ewha Laboratory Animal Genomics Center under specific pathogen-free conditions. The mice were maintained on a 12-h light/12-h dark schedule with *ad libitum* access to food and water. The genotypes for the *Apc* allele were determined by PCR using the following primers: *Apc*^min/+^: forward, 5′-ttccactttggcataaggc, and reverse, 5′-gccatcccttcacgttag; and *Apc*^+/+^: forward, 5′-ttccactttggcataaggc, and reverse, 5′-ttctgagaaagacagaagtta. The genotypes for the *Ino80* allele were determined as previously described [18]. The mice were euthanized using CO_2_ asphyxiation prior to tissue collection. All mouse experiments were approved by the Institutional Animal Care and Use Committee (IACUC) of Ewha Womans University.

### Cell culture and siRNA transfection

The human colorectal cancer cell lines used in this study were purchased from ATCC (Manassas, VA). The CCD-18Co normal colon fibroblast cell line was purchased from the Korea Cell Line Bank (KCLB) (Seoul, Korea). The HCT15, SW620, SW480, SW48, LoVo, and DLD-1 cells were maintained in RPMI 1640 medium supplemented with 10% fetal bovine serum, 100 U/mL penicillin, and 100 μg/mL streptomycin. The HT29 and HCT116 cells were maintained in McCoy’s 5A medium supplemented with 10% fetal bovine serum, 100 U/mL penicillin, and 100 μg/mL streptomycin. The CCD-18Co cells were maintained in Dulbecco’s modified Eagle’s medium supplemented with 10% fetal bovine serum, 100 U/mL penicillin, and 100 μg/mL streptomycin. All cells were maintained at 37°C in a 5% CO_2_ humidified incubator. The siRNA was transfected using Lipofectamine 2000 reagent (Invitrogen). The sense sequence of the Ino80-specific siRNA was 5′-uuaagagugugauuucucauu.

### Polyp counting

To macroscopically assess adenoma formation, mice were killed using CO_2_ asphyxiation when they were 100 days old, which was when they exhibited sufficient tumor volume and tumor numbers. The small intestine and colon were isolated. The small intestine was subdivided into three equal segments (duodenum, jejunum, and ileum), dissected longitudinally, rinsed, spread flat on paper, and photographed using a digital camera. Tumor frequency and size were determined using ImageJ software.

### Histology and immunohistochemistry

Intestinal tissues were sectioned into three equal segments and rolled into a jelly roll before they were fixed in 10% formalin. The fixed tissues were embedded in paraffin, and 4-mm sections were stained using hematoxylin and eosin. All immunohistochemical procedures were performed as previously described [6]. The sections were deparaffinized in xylene and rehydrated in decreasing concentrations of ethanol. Antigen retrieval was performed using sodium citrate buffer. The sections were treated with 3% hydrogen peroxide for 5 min, blocked in PBS containing 1% BSA, and then incubated with primary antibodies followed by biotinylated anti-rabbit secondary antibodies (Vector Laboratories). After the sections were incubated with VECTASTAIN ABC Reagent (Vector Laboratories), they were visualized using DAB chromogen (Vector Laboratories) and counterstained using hematoxylin. The following primary antibodies were used: anti-Ki-67 (Thermo Scientific, RM-9106; 1:200), anti-phospho-Chk1-S345 (Cell Signaling Technology, 2348; 1:25) and anti-phospho-Chk1-S317 (Cell Signaling Technology, 12302; 1:100). TUNEL staining was performed using an ApopTag peroxidase *in situ* apoptosis detection kit (S7100, EMD Millipore) according to the manufacturer’s instructions. For the Ino80 staining of the colon cancer tissue microarray, the anti-Ino80 antibody from Abcam (ab118787) was used (1:400). Images were acquired using an Olympus BX 51 light microscope.

### Immunofluorescence microscopy

The cells used for immunofluorescence microscopy to detect ATR and phospho-Chk1 foci were processed using essentially the same methods that have been previously described [43, 44]. The images were captured using a Carl Zeiss LSM 880 Confocal Laser-Scanning Microscope and processed using ZEN 2.3 software (Carl Zeiss). Anti-phospho-Chk1-S317 (Cell Signaling Technology, 12302; 1:500), anti-ATR (Abcam, ab 2905; 1:500) and anti-γ-H2AX antibodies (Millipore, 05–636; 1:1000) were used.

### TissueFAXS analysis

The sections that were processed for immunostaining were scanned using a digital TissueFAXS imaging system and evaluated using HistoQuest imaging analysis software (TissueGnostics, Vienna, Austria), which is a cell-based staining intensity analysis tool that uses a nuclear cellular identification marker (in this case, hematoxylin). They were then quantitatively analyzed using a given marker that was labeled using a different color (in this case DAB was labeled brown). All evaluations were performed in specific regions of interest (ROI) that were selected in scanned images by manually tracing around the tumoral regions. The mean intensity of the antibody-stained reaction obtained in the ROI measurement was plotted against the mean intensity of hematoxylin and expressed as the percentage of all nucleated (hematoxylin-positive) cells using a scattergram. Positive cells were selected based on cutoff values that were determined using negative controls and the backward and forward gating tool supplied with the software. All of these procedures were performed at Korea Basic Science Institute (KBSI).

### Apoptosis analysis

Apoptosis was analyzed using Annexin V and propidium iodide (PI, Invitrogen) double-staining with a FITC Annexin V Apoptosis Detection Kit I according to the manufacturer’s instructions (556547, BD Biosciences). A total of 1 × 10^6^ cells were washed twice with cold PBS and resuspended in 1 ml of binding buffer. A 100 μl volume of cell suspension (1 × 10^5^ cells) was mixed with 5 μl of Annexin V-FITC and 5 μl of PI. After the mixture was gently vortexed, the cells were incubated in the dark for 20 min at room temperature and then subjected to flow cytometric analysis using a FACS Calibur (BD Biosciences).

### DNA fiber assay

A DNA fiber assay was conducted as previously described [6]. Briefly, cells were treated sequentially with IdU for 10 min and CldU for 30 min (each at 100 μM), which were then incubated in spreading buffer (0.5% SDS, 200 mM Tris-Cl pH 7.4, and 50 mM EDTA) on a Silane-Prep Slide (Sigma-Aldrich; S4651). DNA fibers were then extended by tilting the slide. After fixing the slides in 3:1 methanol/acetic acid, each slide was treated with 2.5 N HCl for 30 min to denature the extended DNA fibers and then incubated with mouse anti-BrdU (for IdU detection) and rat anti-BrdU (for CldU detection) antibodies. After washed with PBS, the slides were incubated first with a mixture of secondary antibodies (Alexa Fluor^®^ 568 rabbit anti-mouse IgG (Invitrogen; A11061) and Alexa Fluor^®^488 chicken anti-rat IgG (Invitrogen; A21470)) at RT for 30 min. They were then blocked for 15 min before being incubated with another mixture of secondary antibodies (Alexa Fluor^®^568 goat anti-rabbit IgG (Invitrogen; A11011) and Alexa Fluor^®^488 goat anti-chicken IgG (Invitrogen; A11039)). The slides were mounted using Vectashield mounting medium. Fluorescence microscopic images were captured using a Carl Zeiss LSM510 scanning laser confocal microscope. The average track lengths of fork progression were calculated by counting only the red-green tracks (ongoing forks) and converting them to base pairs based on the assumption that a 1 μm-long segment of DNA is equivalent to approximately 2.8 kb.

### Immunoblot analysis

An immunoblot analysis was performed using a standard method with cell extracts that were prepared in lysis buffer (50 mM Tris–HCl pH 8.0, 150 mM NaCl, 0.5% sodium deoxycholate, 0.1% SDS, 1% NP-40, 1 mM DTT, 0.5 mM PMSF, 50 μg/ml pepstatin A, 5 μg/ml leupeptin, 5 μg/ml aprotinin, and a phosphatase inhibitor cocktail (Roche)). The following antibodies were used for immunoblotting: p-Chk1-S317 and p-Chk1-S345 (Cell Signaling Technology), actin and Chk1 (Santa Cruz), and Ino80 (Abcam, ab118787). The uncropped full-length images of all immunoblot gels are provided in Supplementary Figure 5.

### Statistical analysis

The significance of differences between groups was evaluated using Student’s *t*-test in Microsoft Excel unless otherwise indicated. The Wilcoxon rank-sum test was used to analyze tumor incidence, and the Mann-Whitney *U* test was used to analyze the mean Ino80 staining intensities in the tissue microarray (GraphPad Prism). Survival was evaluated using the Kaplan–Meier method, and the difference in the median time to death between the groups was determined using log-rank tests (GraphPad Prism). Two-sided *p* values are given, and values less than 0.05 were considered significant.

## SUPPLEMENTARY MATERIALS FIGURES


