# Supplementary material for: INO80 haploinsufficiency inhibits colon cancer tumorigenesis via replication stress-induced apoptosis
Source: Oncotarget. 2017 Dec 6;8(70):115041–53. doi: 10.18632/oncotarget.22984 (PMC5777752; doi:10.18632/oncotarget.22984)
Supplement: Supplementary file 1 [file oncotarget-08-115041-s001.pdf]

INO80 haploinsufficiency inhibits colon cancer tumorigenesis via replication stress-induced apoptosis

SUPPLEMENTARY MATERIALS

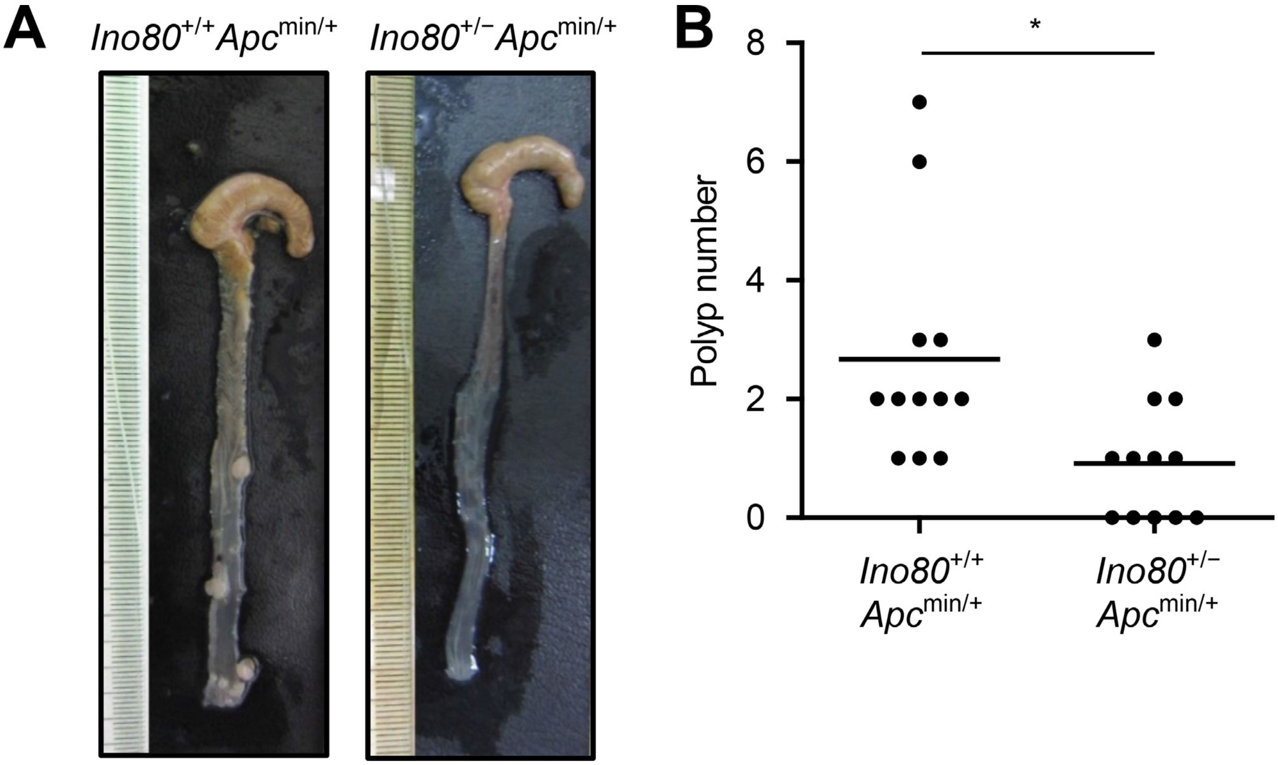

Supplementary Figure 1: *Ino80* haploinsufficiencyinhibits colon tumorigenesis in *Apc*$^{min/+}$mice. (**A**)Representative microscopic images of colonic polyps obtained from the experiments shown in Figure 1a. Magnification, × 40. (**B**)The number of colonic polyps in the indicated mice (n = 12 for each) was plotted as a graph in which each dot represents the total number of polyps in a single mouse. The horizontal bar indicates the mean value. The pvalues were determined using the Wilcoxon rank-sum test. $^{*}p < 0.05$.

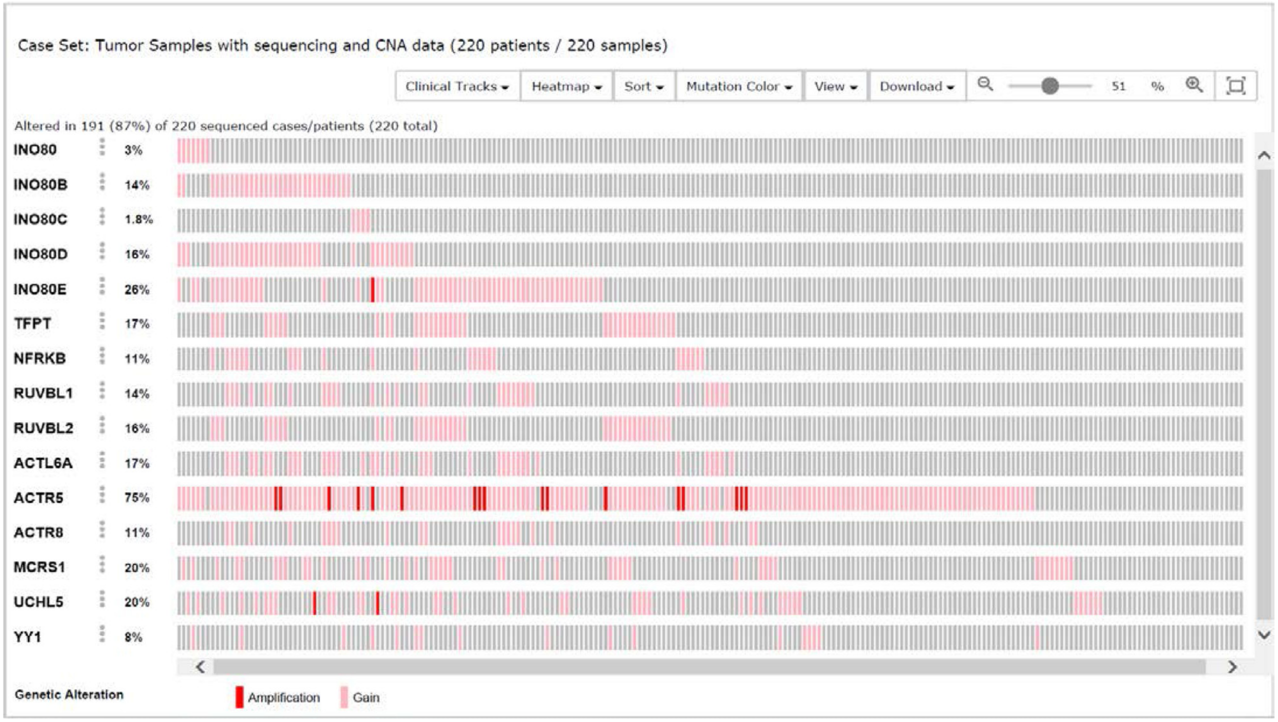

Supplementary Figure 2: Gain and amplification of INO80 subunit genes in colon cancers (TCGA, Provisional; data source, National Institute Cancer). The data were obtained from The Cancer Genome Atlas portal (http://www.cbioportal.org).

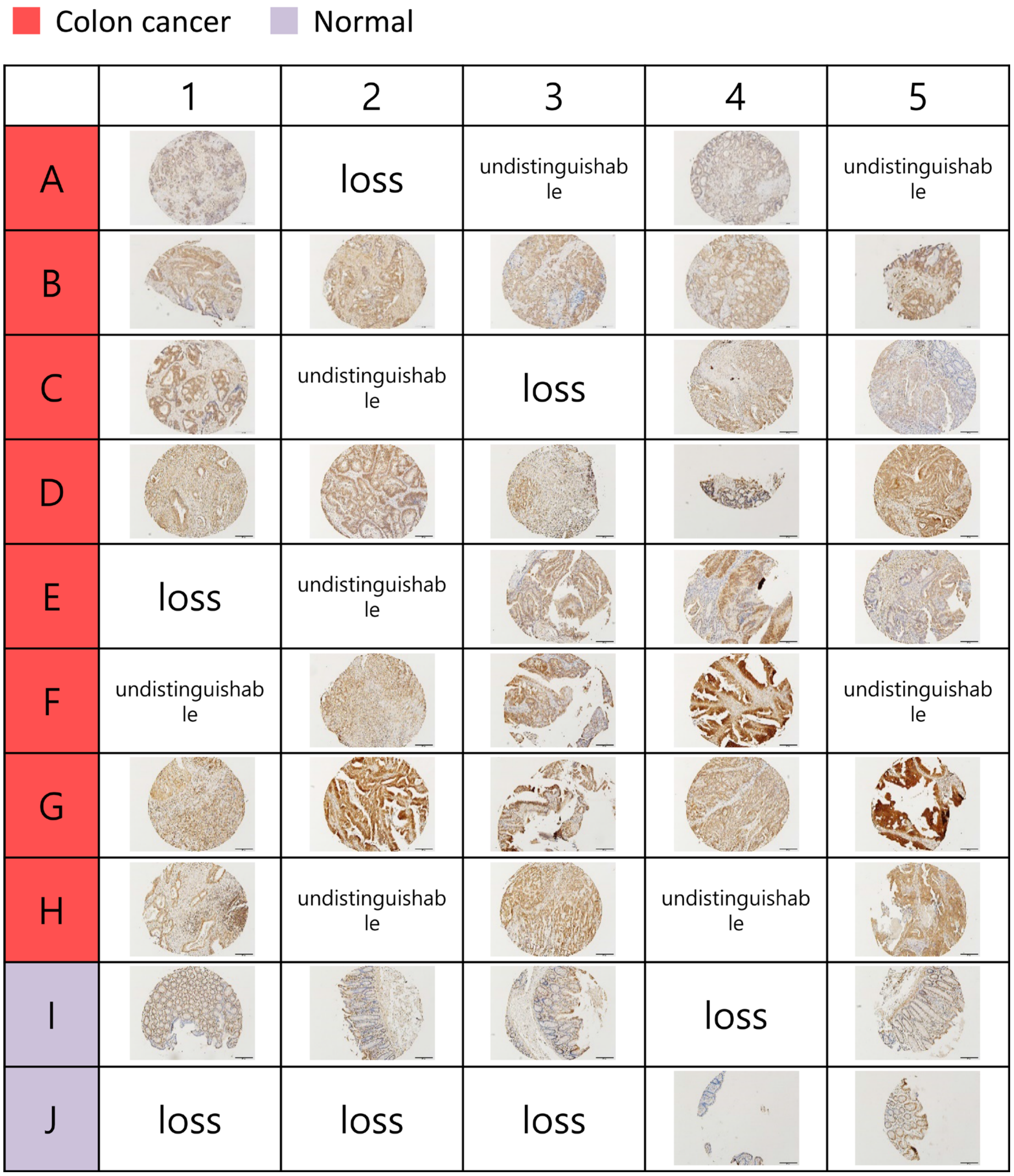

Supplementary Figure 3: Whole images of immunohistochemicalstaining of the colon cancer tissue microarray described in Figure 7C.

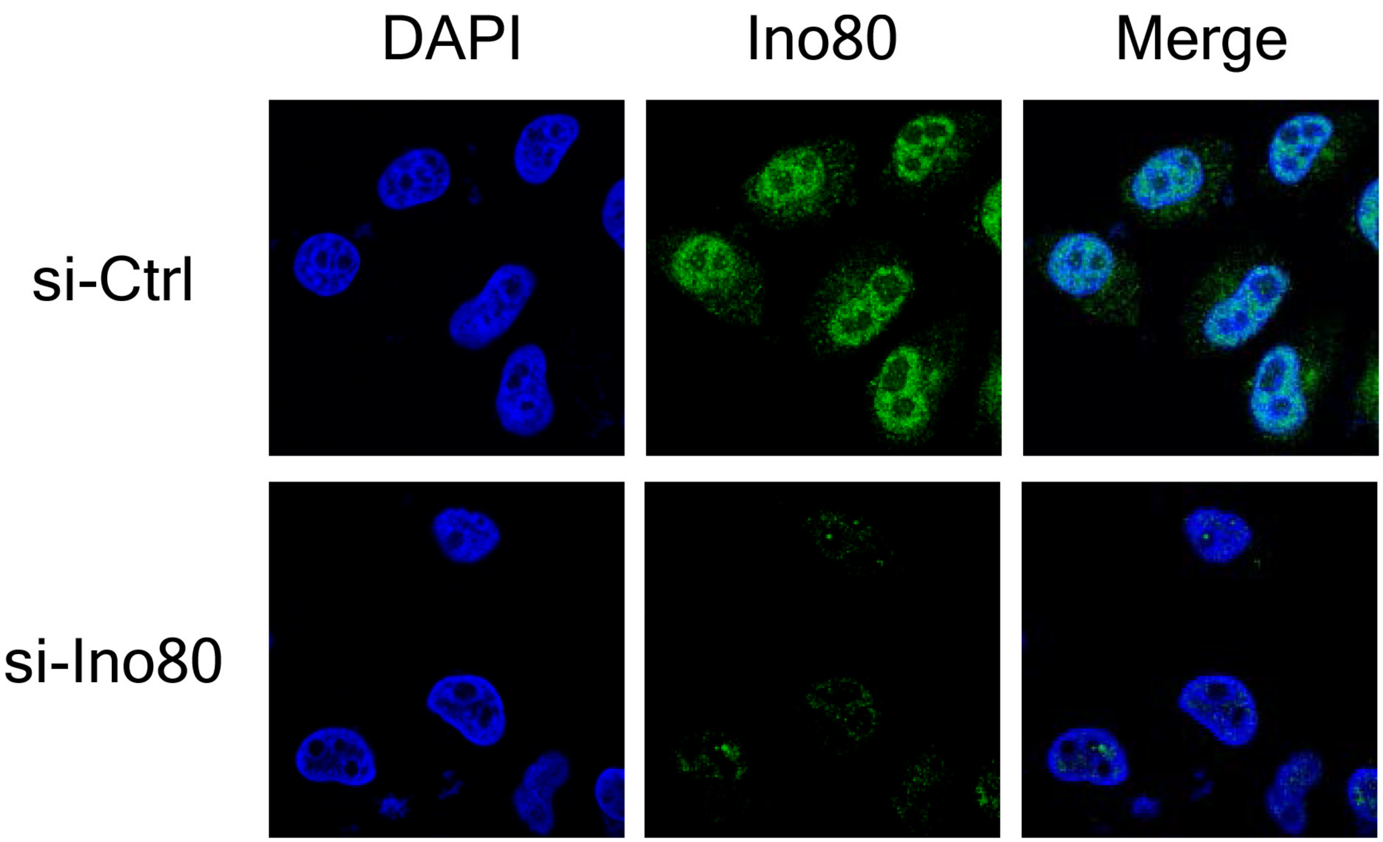

Supplementary Figure 4: Confirmation of the specificity of the anti-Ino80 antibody in immunostaining.HT29 cells were transfected with control or Ino80-specific siRNAs and subjected to immunofluorescence microscopy. The Ino80 signal in the nuclei disappeared after Ino80 knockdown.

Full unedited gel for Figure 3A

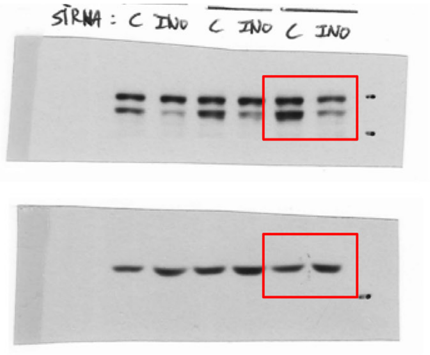

Full unedited gel for Figure 3B

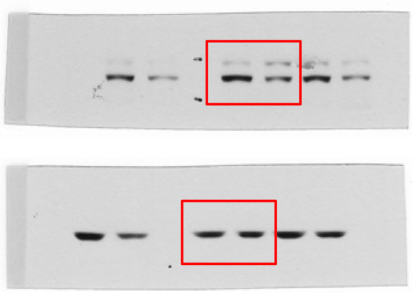

Full unedited gel for Figure 4C

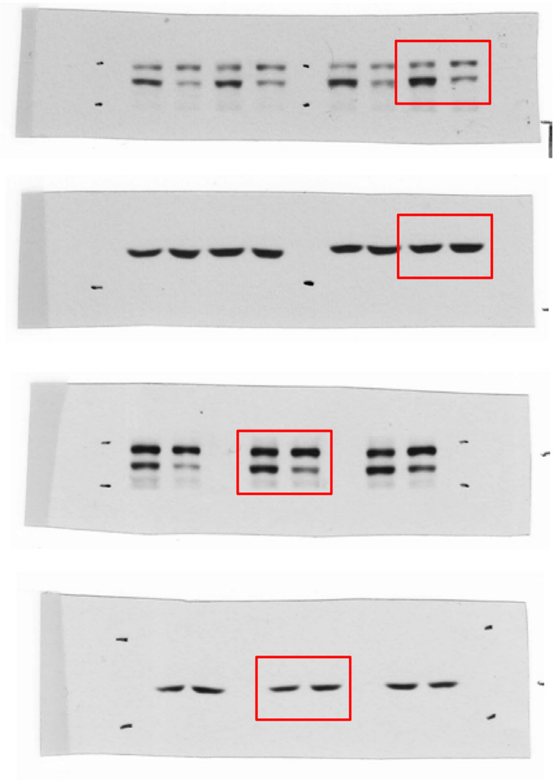

Full unedited gel for Figure 5A

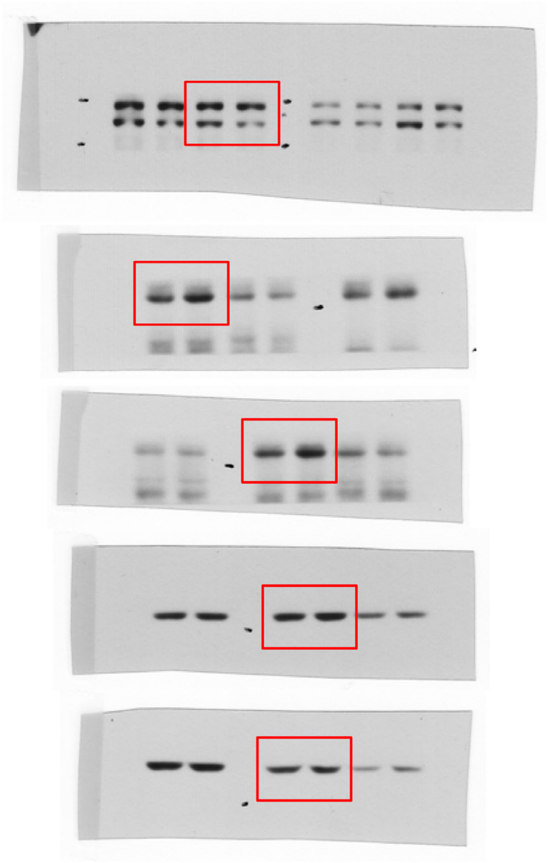

Full unedited gel for Figure 5B

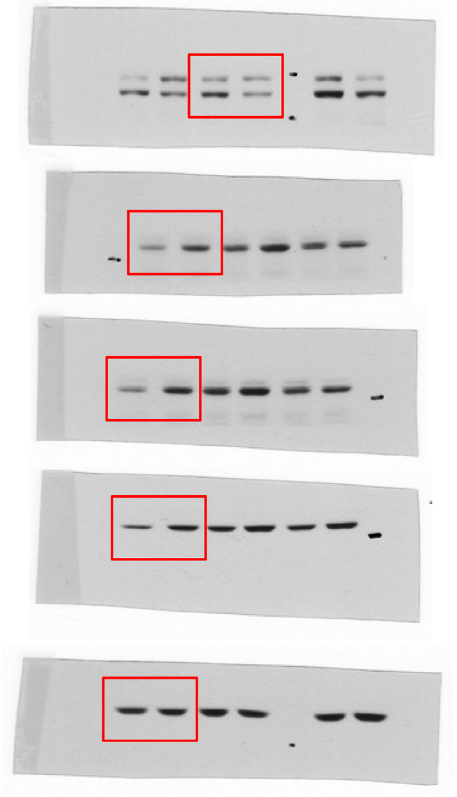

Full unedited gel for Figure 7B

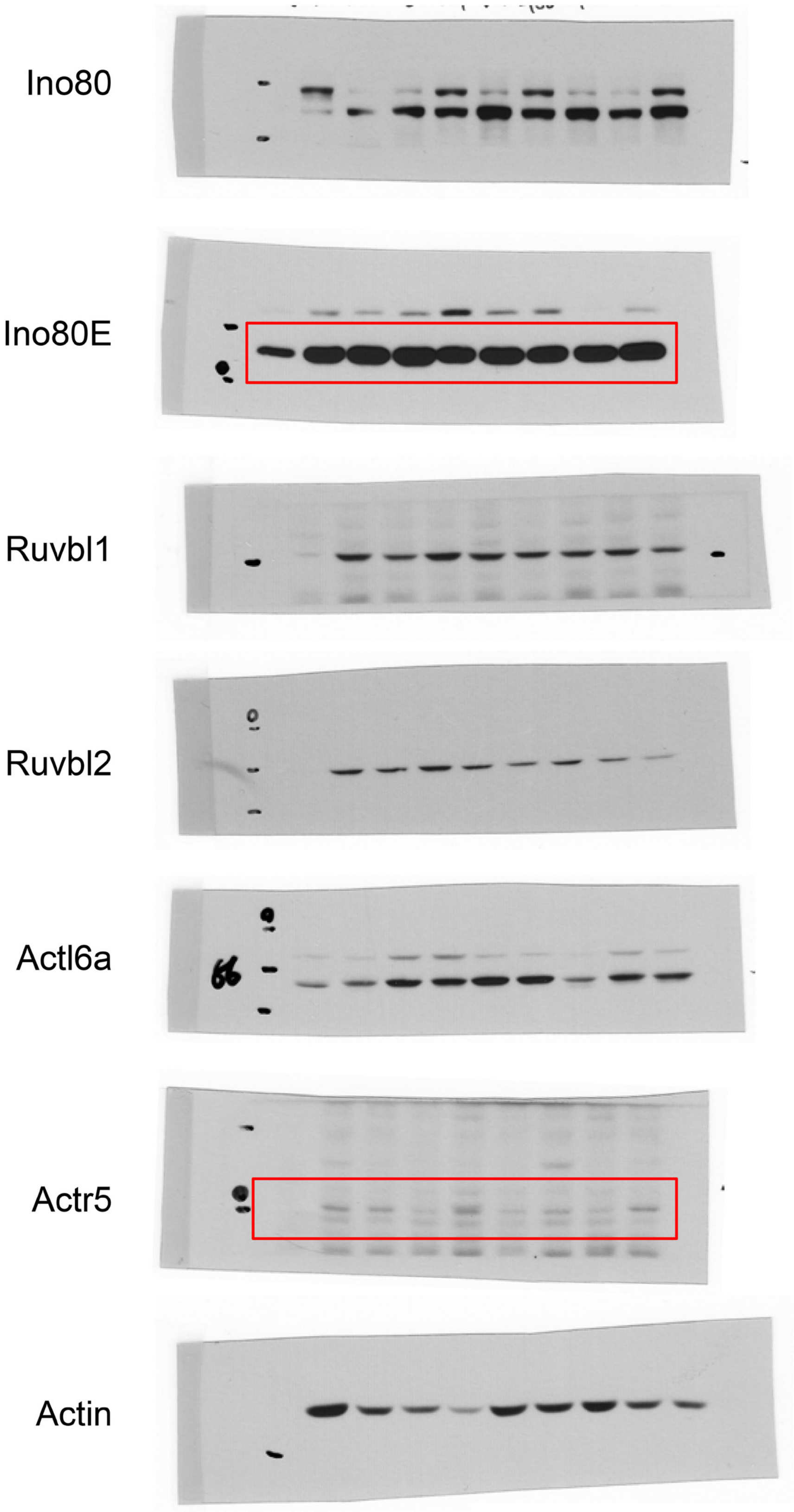

Supplementary Figure 5: Uncropped full-length images of immunoblot gels.
